# The complete chloroplast genome sequence of the *Pueraria lobata* (Willd.) Ohwi (Leguminosae)

**DOI:** 10.1080/23802359.2020.1835576

**Published:** 2020-11-11

**Authors:** Tianxu Cao, Xiling Ma, Yu Zhang, Wenzheng Su, Bicong Li, Qinghong Zhou, Qianglong Zhu

**Affiliations:** Department of Horticulture, College of Agronomy, Jiangxi Agricultural University, Nanchang, PR China

**Keywords:** *Pueraria lobata* (Willd.) Ohwi, kudzu, chloroplast genome, phylogenetic analysis

## Abstract

*Pueraria lobata* (Willd.) Ohwi is an essential traditional oriental medicine with therapeutic effects. In this study, we assembled the complete chloroplast genome of *P. lobata*. The total genome size was 153,442 bp in length, containing a large single-copy (LSC) region of 84,162 bp, a small single-copy (SSC) of 17,998 bp, and a pair of inverted repeats (IRs) of 25,641 bp, and possessing 35.41% GC content. In addition, the whole chloroplast genome encodes a total of 129 genes, including 84 protein-coding genes, 37 tRNA genes, and eight rRNA genes. Phylogenetic tree analysis of 48 species in the family Papilionoideae of Leguminosae indicated that *P. lobata* was belong to Papilionoideae and closely related to the genus, *Pachyrhizus, Vigna* and *Phaseolus*.

*Pueraria lobata* (Willd.) Ohwi is a wild leguminous creeper, commonly known as kudzu or Ge-gen, its swollen fleshy root possesses a high content of puerarin, daidzein, and rutin and is taken as a famous Chinese traditional medicine used to therapy muscle stiffness, common cold, influenza, cardiovascular disease, other ailments (Guerra et al. 2000; Chen et al. 2001; Guo et al. 2020), Kudzu starch has been used as an herb additive in food (Zhu et al. 2018). China is one of the main original and distribution centers of kudzu, but its genetic diversity has been seldom investigated, which seriously hinder germplasm resource conservation, new variety breeding and detection of species invasion (Sun et al. 2005; Yuan et al. 2017). Chloroplast genome was an important part of plant whole genome and play an important role in photosynthesis and other metabolic pathways in kudzu, and the genome sequence is a great molecular resource for genetic diversity, plant taxonomy, and phylogenetic relationships at different taxonomic levels (Ji et al. 2020). So far, about 296 complete chloroplast genomes of Leguminosae have been sequenced and published, but the Chloroplast genome of *P. lobata* has not been reported. Therefore, we reported the complete chloroplast genome sequence of *P. lobata* based on the high-throughput sequencing with hope to promote the studies based on the kudzu chloroplast genome.

The *P. lobata* was collected at Hengfeng, Shangrao, Jiangxi (117°6′E, 28°42′N) in May 2019, and preserved in the Yulan experimental station of Jiangxi Agricultural University, Nanchang, China (Voucher number: JAGE-01). Total genomic DNA was extracted from fresh leaves using modified CTAB (Allen et al. 2006), the high-quality DNA was sent to construct a genomic library and sequenced by BGISEQ-500 platform (BGI, Shenzhen, China). About 3.0 Gb of sequence data was obtained after sequencing and base quality control, 0.5 Gb of clean pair-end reads with length of 150 bp was randomly extracted using Seqtk. The kudzu chloroplast genome was assembled by using SPAdes (v 3.12.0) (Bankevich et al. 2012). Specific method is as follows: first, these reads were assembled with using the Plasmidspades.py in SPAdes, Contigs representing the chloroplast genome were then retrieved, ordered, and incorporated into a single draft sequence by BlastN against the chloroplast genome of *Glycine mix* (CM010429) (Shen et al. 2019), third, the gaps in the chloroplast single draft sequence of were closed by using GapCloser (v1.12-r6). Finally, the complete genome sequence was annotated using CPGAVAS2 (Shi et al. 2019), and manually checked and corrected by Sequin. The annotated genomic sequence was submitted to GenBank under accession number MT818508.

The complete chloroplast genome of *P. lobata* is 153,442 bp in length, and exhibits a typical quadripartite structure, consisting of a pair of IRs (25,641 bp) separated by the LSC (84,162 bp) and SSC (17,998 bp) regions. The LSC, SSC, IR regions and GC contents of the whole chloroplast genome are 34.7%, 28.90%, 41.88%, and 35.41%, respectively. A total of 129 genes are encoded, including 86 protein-coding genes, 8 rRNA genes, and 37 tRNA genes; 6 of the protein-coding genes, 6 of the tRNA genes, and 4 rRNA genes are duplicated within the IRs. Moreover, 11 protein-coding and 6 tRNA genes contained one intron, and 3 protein-coding genes contained two introns respectively. Noticeably, the start code of *ndhD* is by RNA editing, and an exon of rps12 is by trans-splicing.

To date, the complete chloroplast genomes of 47 genera of Papilionoideae have been reported at GenBank, to confirm the phylogenetic location of *P. lobata*, a phylogenetic analysis was conducted using common 63 chloroplast protein-coding sequences of *P. lobata* and other 47 published chloroplast genomes represented different genus. These chloroplast protein-coding sequences were aligned by the MAFFT v7.407 (Nakamura et al. 2018), the phylogenetic tree was constructed by maximum-likelihood method with 1000 bootstrap value using MEGA v10.0.4 (Kumar et al. 2018). The phylogenetic analysis results clearly showed that *P. lobata* was closer to *Pachyrhizus* (*P. erosus*), *Vigna* (*V. angularis*), and *Phaseolus* (*P. vulgaris*), but keep remote phylogenetic relationship with these genera, *Lathyrus*, *Trifolium*, and *prisum* in the family Leguminosae (Figure 1). The chloroplast genome of *P. lobata* will provide useful genetic information for promoting the evolutionary studies and conservation of Leguminosae species.

**Figure 1.**
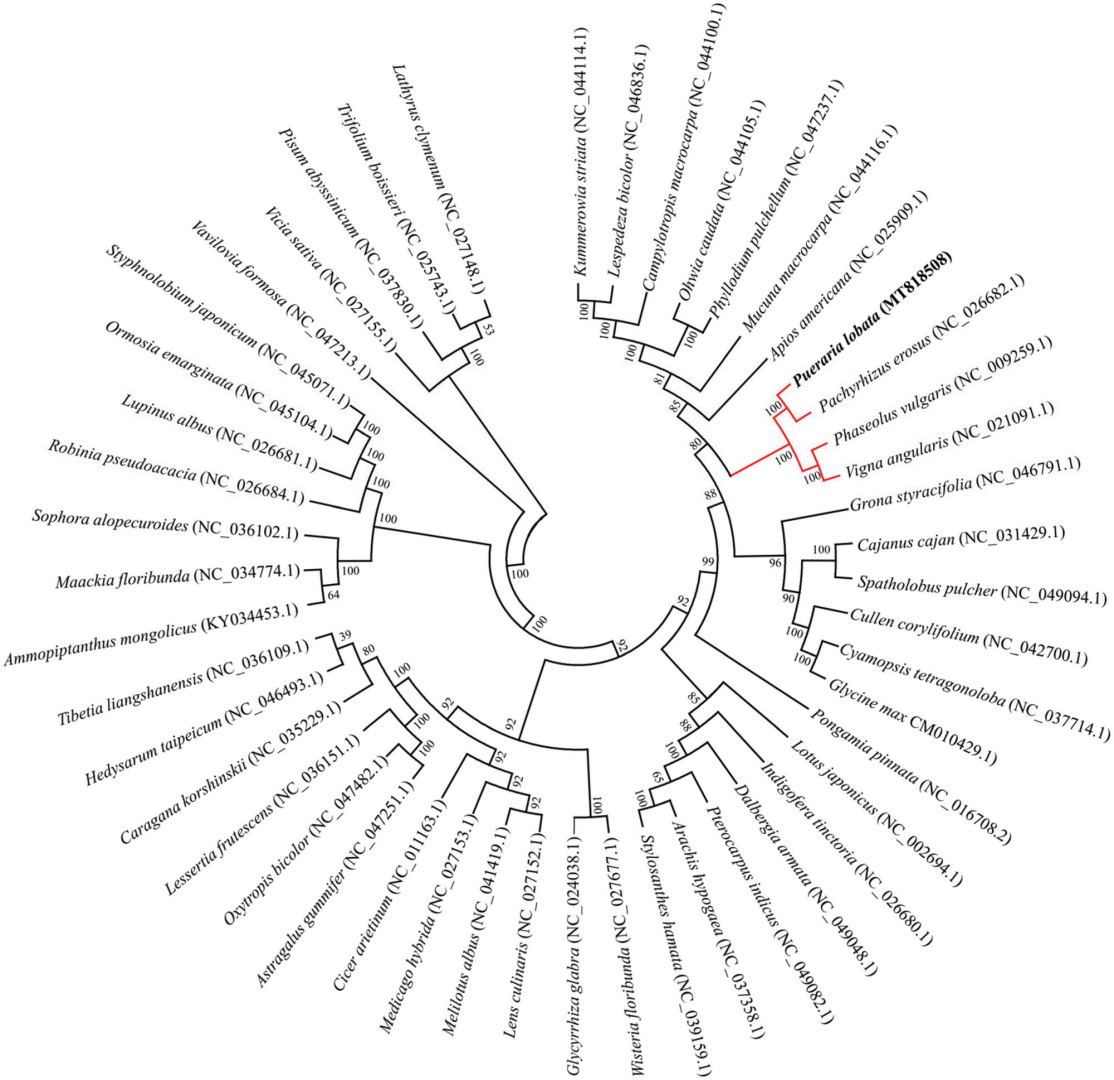
Phylogenetic tree showing relationship between *P. lobata* and 48 species belonging to Papilionoideae in Leguminosae family. Phylogenetic tree was constructed based on 63 protein-coding genes of chloroplast genomes using maximum likelihood (ML) with 1000 bootstrap replicates. Numbers in each the node indicated the bootstrap support values.

## Data Availability

The data that support the findings of this study are openly available in GenBank at https://www.ncbi.nlm.nih.gov, reference number MT818508.
